# Individual and interactive effects of white-tailed deer and an exotic shrub on artificial and natural regeneration in mixed hardwood forests

**DOI:** 10.1093/aobpla/plx024

**Published:** 2017-06-08

**Authors:** Charlotte F. Owings, Douglass F. Jacobs, Joshua M. Shields, Michael R. Saunders, Michael A. Jenkins

**Affiliations:** 1 Department of Forestry and Natural Resources, Purdue University, 715 West State Street, West Lafayette, IN 47906, USA; 2 Manistee Conservation District, 8840 Chippewa Highway, Bear Lake, MI 49614, USA

**Keywords:** Ecological restoration, field experiment, forest development, herbivory, invasive plants, moisture stress, natural and artificial regeneration, ungulates

## Abstract

Underplanting tree seedlings in areas where natural regeneration is limited may offer a tool by which desired overstory composition can be maintained or restored in forests. However, invasive plant species and ungulate browsing may limit the effectiveness of underplanting, and in-turn, the successful restoration of forest ecosystems. Individually, the invasive shrub *Lonicera maackii* and browsing by white-tailed deer (*Odocoileus virginianus*) have been found to negatively affect the regeneration of native tree species in the Midwestern United States, but few studies have examined their interactive or cumulative effects. Using exclosures and shrub removal at five sites, we examined the effects of white-tailed deer and *L. maackii* both on underplanted seedlings of *Castanea dentata* and *Quercus rubra* and on the composition, species richness and diversity of naturally regenerated native tree seedlings. Individually, both deer and *L. maackii* had negative effects on the survival of underplanted seedlings, but we identified no interactive effects. The presence of *L. maackii* or deer alone resulted in similar declines in the survivorship of *Q. rubra* seedlings, but the presence of deer alone resulted in lower survival of *C. dentata* seedlings than the presence of *L. maackii* alone*. Lonicera maackii* reduced light levels, increased seedling moisture stress and decreased relative basal diameter growth for *Q. rubra* seedlings. Deer reduced the relative growth in height of underplanted *C. dentata* and *Q. rubra* seedlings and increased moisture stress of *C. dentata* seedlings. No effects of *L. maackii* or deer were found on soil or foliar nitrogen or the overall abundance, species richness and diversity of naturally regenerated seedlings. However, *L. maackii* and white-tailed deer did affect the abundance of individual tree species, shifting composition of the regeneration layer towards shade tolerant and unpalatable and/or browse tolerant species.

## Teaser

We experimentally excluded deer and removed an invasive shrub (Amur honeysuckle) to determine their effects on the survival of planted tree seedlings and the abundance and diversity of natural seedlings. The presence of honeysuckle or deer alone resulted in similar declines in the survivorship of planted oak seedlings, but the presence of deer alone resulted in lower survival of planted chestnut seedlings than the presence of honeysuckle alone. While deer and honeysuckle did not affect the abundance or diversity of natural seedlings, the presence of both factors increased the dominance of shade-tolerant, unpalatable, and/or browse tolerant species.

## Introduction

Successful regeneration of overstory species is integral to maintaining forest systems in a time of ecological change (Reyer *et al.* 2015). However, overabundant ungulate populations and the spread of invasive plants pose a threat to the regeneration of ecologically and economically valuable native tree species in many parts of the world (Coomes *et al.* 2003; Vavra *et al.* 2007; Jacobs *et al.* 2015). The negative effects of ungulates and invasive plants are typically more pronounced in fragmented landscapes, where forests exist as small patches within a matrix of agriculture and exurban development (Minor *et al.* 2009; Hurley *et al.* 2012). Within North America, the Midwestern United States offers an archetype of a fragmented landscape altered by invasive plants (Luken 1997; Oswalt *et al.* 2015) and a frequently overabundant ungulate species (white-tailed deer; Anderson 1997; Hurley *et al.* 2012).

In the last century, white-tailed deer abundance has increased as a result of reduced predation, greater forage from agriculture and tree plantings and increased edge habitat (Côté *et al.* 2004). White-tailed deer preferentially browse certain species, altering forest dynamics over time by shifting composition towards species that are unpalatable or browse-tolerant (Rooney and Waller 2003; Rossell *et al.* 2005). White-tailed deer alter nutrient cycling by preferentially browsing plants that have nutrient-rich tissue, over time, increasing the abundance of nutrient-poor species which decompose more slowly (Ritchie *et al.* 1998; Côté *et al.* 2004). White-tailed deer alter nitrogen cycling by increasing the amount of available nitrogen in the soil through faeces and urine and by altering plant composition, and thus litter quality, through herbivory (Hobbs 1996; Ritchie *et al.* 1998; Murray *et al.* 2013). Ultimately, composition and structure of forests are altered as heavily browsed species such as oak (*Quercus* spp.) are lost, and the regeneration layer is dominated by less-preferred or browse-tolerant species such as *Fraxinus americana* or *Prunus serotina* (Tilghman 1989; Rossell *et al.* 2005).

In addition to ungulates, natural regeneration of native tree seedlings can be limited by competition with other species, in particular invasive plants. Amongst invasive plants species, shrubs are often the most problematic in forests because they form thick understory layers that are alien structural elements in many forests and overtop woody seedlings (Merriam and Feil 2002; Webster *et al.* 2006; Shields *et al.* 2015a). For example, one species of invasive shrub that limits the natural regeneration of native tree seedlings is *Lonicera maackii* (Hutchinson and Vankat 1997; Collier *et al.* 2002; [Bibr plx024-B27][Bibr plx024-B73]), which was first cultivated in the United States in the late 1800s and has now spread to 28 states (Luken and Thieret 1994; USDA, NRCS 2015). *Lonicera maackii* is a superior competitor to many native species, grows rapidly, has extensive roots near the soil surface that facilitate the uptake of nutrients and water, and possesses an extended leaf phenology compared with native species (Gould and Gorchov 2000; Hutchinson and Vankat 1997; McEwan *et al.* 2010; Pfeiffer and Gorchov 2015). In addition, *L.maackii* has been found to alter nutrient cycling in invaded areas through accelerated release of litter N resulting from a lower C:N ratio and more rapid litter decomposition than native species (Blair and Stowasser 2009; Poulette and Arthur 2012; Schuster and Dukes 2014). These alterations could align the release of nitrogen with the expanded growing season of invasive shrubs such as *L. maackii*, potentially furthering its competitive advantage (Blair and Stowasser 2009; Schuster and Dukes 2014).

*Quercus rubra* and *Castanea dentata* are two economically and ecologically valuable tree species that have limited natural regeneration across their extensive historic range in eastern North America. *Quercus rubra* has experienced widespread regeneration failure as a result of fire suppression (Brose *et al.* 2014) and herbivory by overabundant populations of white-tailed deer (Buckley *et al.* 1998; Castleberry *et al.* 1999; Rooney and Waller 2003), resulting in drastic increases in the dominance of shade- and browse-tolerant mesophytic species in forest understories (Abrams 1992; Brose *et al.* 2001, 2014). *Castanea dentata* has largely disappeared from its historically extensive range as the result of chestnut blight (*Cryphonectria parasitica*), a pathogen introduced in the early 1900s (Paillet 2002). The loss of this foundation species spurred research efforts to develop a blight-resistant hybrid though backcross breeding with *Castaneamollissima*, an Asian species (Jacobs *et al.* 2013). The resulting seedlings are primarily *C. dentata* genetically (>90 %) and may offer an opportunity to restore *C. dentata* to native forests as restoration prescriptions are developed (Burnham 1986; Diskin *et al.* 2006; Jacobs *et al.* 2013).

In the long-term, restoring degraded forests in fragmented landscapes may depend upon artificial regeneration techniques, such as underplanting, to increase the importance of desired species in hardwood forests (Dey *et al.* 2012). Underplanting can reduce the amount of site preparation necessary for restoration, thus decreasing the amount of resources needed to restore forested areas (Belair *et al.* 2014). Underplanting can be used in areas with low abundance of natural regeneration and may allow management to function on controlled time scales as the technique does not require the long process of fostering natural regeneration (Loftis 1990; Paquette *et al.* 2006). For species such as *Q. rubra*, seedling growth is optimized under partial canopy (often created by shelterwood harvest) and the underplanting of seedlings can provide advanced regeneration before initial openings are created (Loftis 1990). Recent work has shown that the growth of *C. dentata* seedlings exhibit a similar response to partial canopy removal and outperform other species, including *Q. rubra*, when underplanted (Belair *et al.* 2014). While underplanting is a useful restoration tool, obstacles such as herbivory from wildlife and competition from other species may reduce the successful establishment and growth of underplanted seedlings (Dey *et al.* 2012).

While the impacts of *L. maackii* and white-tailed deer on native tree seedlings have been studied individually, less is known about their combined effects. *Lonicera maackii* in the understory could help to physically protect tree seedlings from deer browsing by providing cover, but may also create a microenvironment that inhibits the germination, establishment, growth and survival of seedlings. Aronson and Handel (2011) suggested that management of invasive plants and white-tailed deer may be necessary for the natural regeneration of native canopy trees to persist. In a study of the effects of honeysuckle and white-tailed deer on the survival of sugar maple seedlings, Loomis *et al.* (2015) found that, individually, both honeysuckle and white-tailed deer negatively affected seedling survival, but no significant interaction between the two factors was found. Active management methods, such as underplanting, protecting planted tree seedlings from herbivory and removing invasive shrubs may be necessary to restore forested areas in which both invasive shrub species and white-tailed deer are present.

In this study, our objectives were to examine the individual and combined impacts of *L. maackii* and white-tailed deer on underplanted *Q. rubra* and *C. dentata* seedlings, as well as on the composition of naturally regenerated seedlings of woody species. We conducted our study in five forests within the glaciated till plain of IN, USA (Table 1). Typical of the Midwest region, forests in this area largely consist of small fragments within an agricultural matrix. Deer hunting occurred on three of our sites and in the matrix surrounding the remaining two sites. We did not observe evidence of heavy deer browse (browse lines, lack of native woody understory in areas without dense *L. maackii*, extirpation of lily species; Waller and Alverson 1997) at any of our sites. For our study design, we utilized two treatments: deer exclusion and honeysuckle removal.

**Table 1:** Locations, average annual precipitation, dominant overstory species, soil type and age of invasion for five study sites in IN, USA. The dominant overstory species were obtained from Shields et al. (2015b), soil type information was taken from the USDA web soil survey (Natural Resource Conservation Service 2016), average annual precipitation was calculated from 1981 to 2010 period (NOAA), and age of invasion was determined from counting the rings of stem cross-sections from *L. maackii* shrubs harvested to create the removal areas (Shields et al. 2014). The age of invasion at Martell was determined using a linear mixed effects model and harvested stem cross sections to create an age model to predict the age of the oldest *L. maackii* shrub (Shields et al. 2014). *Lonicera maackii* density (mean ± 1 SE is for stems > 1.37 m tall. Deer visits represent the combined number of deer photographed by four cameras for a total of four weeks (two weeks in June and two weeks in September 2014) for each study site.

Study site	Lat/long	Annual precip. (cm/yr)	Dominant overstory species	Soils	Invasion age (years)	*L. maackii* density (stems/ha)	Deer visits
Lugar Farm	40° 25′N 86° 57′W	97.03	*Robinia pseudoacacia*, *Juglans nigra*	Silt loams	35	3135 ± 863	19
Martell	40° 26′N 87° 01′W	97.03	*Quercus alba*, *Quercus velutina*	Silt loams	13	854 ± 1677	47
Pursell	40° 17′N 86° 52′W	98.83	*Maclura pomifera*, *Prunus serotina*	Loamy sands Sandy loams Silt loams	23	1354 ± 1249	54
Ross	40° 24′N 87° 04′W	98.83	*Q. velutina*, *Liriodendron tulipifera*	Loams Sandy loams Silt loams	18	1042 ± 1134	86
Terre Haute	39° 21′N 87° 26′W	111.35	*L. tulipifera*, *P. serotina*, *Sassafras albidum*	Fine sandy loams	30	2375 ± 773	34

We hypothesized that white-tailed deer and *L. maackii* would collectively have more negative impacts on forest seedlings than either treatment alone. However, when both treatments were considered individually, we further hypothesized that *L. maackii* would have a greater suppressive effect on seedlings than deer, but would also mitigate the direct effects of deer. Specifically, due to above and below competition created by high stem densities of *L. maackii* in invaded sites, we predicted (i) growth, survival and foliar nitrogen content of underplanted seedlings would be lower and water stress higher in the presence of *L. maackii* alone than in the presence of deer alone. However, we predicted that (ii) the rate of browsing of underplanted seedlings outside of the exclosures would be lower under the protective cover of *L. maackii.* We also predicted that (iii) acute competition created by the presence of *L. maackii* alone would result in lower abundance, richness and diversity of naturally regenerated seedlings than in the presence of deer alone. 

## Methods

### Study areas

This study was conducted at five sites within the glaciated region of IN, USA: (i) Martell Experimental Research Forest (Martell); (ii) Vigo County Park District property (Terre Haute); (iii) Ross Biological Reserve (Ross); (iv) Purdue University Department of Forestry and Natural Resources Lugar Farm (Lugar Farm) and (v) a privately owned woodlot (Pursell; Table 1). Sites were within mature secondary deciduous forests that were heavily invaded by *L. maackii* and in which *L. maackii* was the dominant invasive species (Shields *et al.* 2015a). The age and level of *L. maackii* invasion, soil type and overstory composition varied across the five sites (Table 1).

Two 80 × 80 m areas were designated at each of the five study sites. In one 80 × 80 m area, all woody invasive plant species were removed between November 2010 and March 2011 at four sites (Terre Haute, Ross, Lugar Farm and Pursell) and in February 2013 at the remaining site (Martell). The second 80 × 80 m area was untreated and served as a reference area (Shields *et al.* 2015b). *Lonicera maackii* was removed by either cutting the shrub at the base using a brush saw or loppers and treating the stump with herbicide (20 % Garlon 4^®^ triclopyr, 1 % Stalker^®^ imazapyr and 79 % Ax-it^®^ basal oil), or by manually pulling small shrubs (single stems < 80 cm height) out of the ground (Shields *et al.* 2015b). After cutting, large shrubs were then removed from the site. New and re-sprouted shrubs were re-treated by cutting and applying herbicide on the cut surface in the summer of 2014 to maintain the removal areas.

In the spring of 2013, two 20 × 40 m units were established in each 80 × 80 m removal and reference area. One of the two units was randomly selected as a deer exclosure area and a 2.5-m tall fence was constructed around the exterior to prevent white-tailed deer from accessing the unit. Small mammals, however, were able to enter the exclosure beneath the fence. Fences were checked periodically for damage and repaired as necessary. No fence was constructed around the second 20 × 40 m unit, allowing deer to access these areas. After fence construction, each study site contained four treatment combinations: (i) *L. maackii* removed and accessible to deer, (ii) *L. maackii* removed and deer excluded, (iii) *L. maackii* present and accessible to deer and (iv) *L. maackii* present and deer excluded.

A severe windstorm on 17 November 2013 resulted in heavy damage to the removal area at the Lugar Farm site. The storm resulted in windthrow of over half the forest canopy, resulting in increased light availability and large inputs of woody debris. Debris was cut and removed from around the planted seedlings and natural regeneration transects in the two removal subunits to allow deer access comparable to the pre-storm condition. In the reference area, which was largely undamaged, selected trees were girdled in the spring of 2014 in order to create similar openings in the canopy to those in the removal areas while preserving the dense *L. maackii* shrub cover.

### Tree seedling study species and underplanting

Each treatment unit was divided length-wise into two sections. One of the sections was randomly assigned for tree seedling planting and the other was assigned for the sampling of natural regeneration. For planting, we obtained 800 one-year-old bareroot *Q. rubra* and *C. dentata* seedlings that were produced according to standard operational nursery practices (Jacobs 2003) at Vallonia State Tree Nursery in southern Indiana. Twenty seedlings of each species were planted by hand in two lines spaced 2 m apart in each study unit in April of 2014. Within lines, the two species were randomly mixed and seedlings were planted 1.5 m apart. To allow establishment, competing vegetation was removed by hand within a meter of each seedling at the beginning of the study.

### Seedling characteristics and browse

Survival, height and basal diameter (to the nearest 0.01 mm) were measured for each seedling at the beginning and end of the growing season. The presence of deer and rabbit browse was also recorded. Rabbit browse was distinguished from deer browse by examining the browsed area on the stem of the underplanted seedling. Rabbit browsed seedlings had clean and angled cuts while seedlings browsed by deer were more scraped and jagged in appearance. To confirm the presence of deer at each of the sites, two trail cameras (HC600 Hyperfire, RECONYX, Inc., Holmen, WI, USA) were placed in the two non-exclosure (removal and reference outside) subunits at each of the study sites during June and September 2014 and images were examined for deer. Total deer visits ranged from 19 at Lugar Farm to 86 at Ross (Table 1).

### Seedling moisture stress and foliar nutrient concentration

Pre-dawn plant moisture stress was measured for five randomly selected seedlings per species from each of the units during 12–15 August 2014. One leaf was collected from each seedling at approximately the same location along the stem and the leaf xylem water potential was determined using a pressure chamber (Model 600, PMS Instruments, Corvallis, OR, USA; Waring and Cleary 1967).

After moisture stress measurements were taken, collected leaves were dried at 65 °C for 48 h. The dried samples were then ground in a ball mill. Foliar N concentration was determined for each foliar sample using an elemental analyzer (ECS 400, Costech Analytical Technologies, Inc., Valencia, CA, USA).

### Light measurements and soil N availability

To determine the amount of photosynthetically active radiation (PAR) in each study unit, PAR measurements were taken using a light ceptometer (LP-80 AccuPAR Ceptometer, Decagon Devices, Inc., Pullman, WA, USA) in July 2015. All measurements were made ∼1 m above the ground on cloudless days between 1 h prior and 1 h after solar noon. PAR measurements were taken in an open field adjacent to each study site to determine the ambient PAR (Grayson *et al.* 2012). Within the study units, PAR readings were taken above every fifth underplanted seedling.

Twelve soil subsamples from 0 to 20 cm depth were collected from each of the four treatment subunits using a soil probe. Four composite samples were formed by pooling three soil samples randomly chosen from the same subunit. Samples were refrigerated and then sent to Brookside Laboratories, Inc. (New Bremen, OH, USA) and tested for total nitrogen (NO_3_ and NH_4_; Nelson and Sommers 1996; McGeehan and Naylor 1988).

### Natural tree regeneration

In the section of the treatment unit assigned to natural regeneration, three 10 m-long permanent transects were established with each transect spaced a minimum of 5 m from the next nearest transect. Five 1 m^2^ quadrats were placed every other meter along the right side of each 10 m transect. Woody stems <50 cm in height were tallied by species within the 1-m^2^ quadrats during late July/early August 2013, 2014 and 2015. The stem and species tallies were used to determine the density, species richness and species diversity of naturally regenerating woody stems for each unit. Woody vines were not included in the tally.

### Calculations and statistical analyses

For each underplanted seedling, relative changes in height and basal diameter were calculated by determining the change in height and basal diameter over the course of the growing season (height/basal diameter in fall – height/basal diameter in spring) and dividing this value by the initial height and initial basal diameter, respectively. For the relative change in height and basal diameter analyses, dead trees were excluded. Negative values of relative growth in basal diameter were excluded from analyses as likely being a reflection of dead seedlings. Relative changes in growth data (height and basal diameter) were transformed using an arcsine square root transformation to improve normality. Percent ambient PAR was calculated by dividing the PAR value recorded above every fifth seedling by the average ambient PAR value from 15 readings (Grayson *et al.* 2012). These values were log-transformed to improve normality for statistical analyses. The species richness, evenness and diversity of naturally regenerating seedlings were calculated from the natural regeneration quadrat data. Species richness was calculated as the number of unique species per transect and species diversity was calculated using the Shannon Diversity Index.

Species diversity, evenness and richness were calculated using the software PC-ORD 5 (McCune and Mefford 2011). Species within the same genus were grouped together for analysis because many individual species did not occur across all study sites. These groupings included *Carya* spp., *Quercus* spp. and *Ulmus* spp.

All statistical analyses were performed using R statistical software (R Core Team 2013) and significance was determined at *α* = 0.05. The R statistical packages ‘survival’ and ‘coxme’ (Therneau 2015) were used to determine the fixed effects of *L. maackii*, white-tailed deer, their interaction, and the random effect of site on the survival of the underplanted *C. dentata* and *Q. rubra* seedlings. The ‘coxme’ and ‘survival’ packages were used to analyse survival using a Cox proportional hazards model to determine differences in the relative risk of mortality amongst treatments from the beginning to the end of the study.

Generalized linear mixed effects models were performed using the R package ‘lme4’ (Bates *et al.* 2015) to determine the fixed effects of *L. maackii*, white-tailed deer, their interaction, and the random effect of study site on foliar and soil nitrogen concentration, moisture stress, browse, natural regeneration, PAR and underplanted seedling growth (basal diameter and height). Time (year of measurement) was also included as a factor in the natural regeneration analyses as these data represented changes across three years (2013, 2014 and 2015) while the data for the underplanted seedlings represented change over one year or one time point of measurement. Each site was considered as a replicate in our analyses. A Poisson distribution was used to model natural regeneration density, an inverse Gaussian distribution was used to model moisture stress, and all other measurements were modelled using a normal distribution. Data from Ross were excluded from the plant moisture stress and foliar N analyses as one of the treatments had no trees with foliar tissue to measure. However, we were still able to compare plant moisture stress and foliar N across all four treatments at the other four sites (Farm, Martell, Pursell and Terre Haute).

Because of the large number of statistical tests performed in our comparisons of natural regeneration density by species, we adjusted *P*-values (*q*-values, Pike 2011) with a graphically-sharpened procedure based on control of the false discovery rate (FDR; Benjamini and Hochberg 1995, 2000). In recent years, multiple comparison techniques based upon FDR have been used more frequently in ecological experiments as an alternative to traditional controls of family-wise error rate because FDR-based techniques retain statistical power while keeping the proportion of false discoveries small relative to all significant results (Verhoeven *et al.* 2005; Pike 2011).

## Results

### Survival of underplanted seedlings

**Figure 1.**
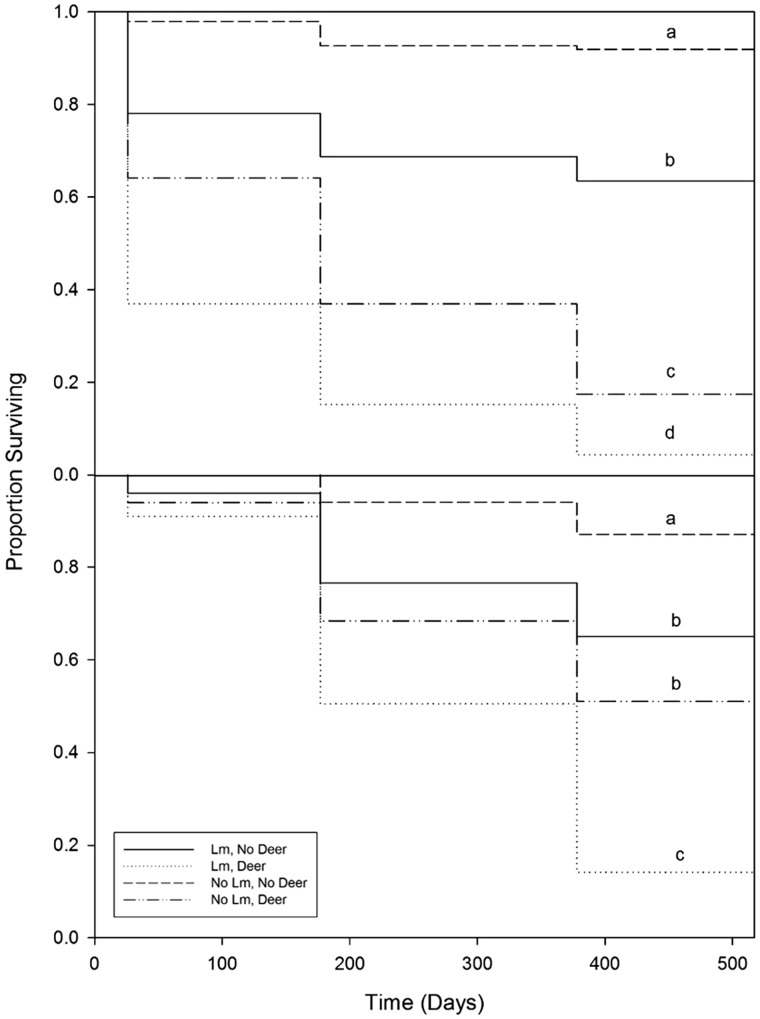
Proportion of surviving *C. dentata* (top) and *Q. rubra* (bottom) seedlings over time across treatments with *L. maackii* (Lm), without *L. maackii* (No Lm) and with white-tailed deer (Deer) and where white-tailed deer were excluded (No Deer). Data represent actual data points, with different lowercase letters designating significant differences in probability of mortality between treatments.

Supporting our hypothesis, the greatest survival for both species was in the treatment combination where *L. maackii* was removed and deer were excluded, while the lowest survival for both species was in the reference areas outside of the exclosures where only ∼10 % of seedlings survived after two growing seasons (Fig. 1, Table 2). There was no significant interaction between *L. maackii* and white-tailed deer for the survival of either species. For *C. dentata*, the second highest survival rate was in areas in which *L. maackii* was present and deer were excluded, countering our prediction that *L. maackii* alone would have a greater impact on survival than deer alone. *Lonicera maackii* removal areas outside the exclosures exhibited the next lowest survival rate. For *Q. rubra*, survival did not differ between the treatment in which *L. maackii* was present inside the exclosures and where it was removed outside the exclosures (Fig. 1).

**Table 2:** Mixed model results for the effects of *L. maackii* (LM) and deer (D) on survival, browse, relative change in height and basal diameter, plant moisture stress, foliar N and environmental variables. Values represent *P*-values for the main effects and main effect interactions with values in bold representing significant effects (*P* < 0.05). Chi square values are reported for survival because a Cox proportional hazards model was used for this analysis (please see methods section). Generalized linear mixed effects models were used for all other analyses.

		*C. dentata*		*Q. rubra*	
Seedling characteristic		Chi sq.	*P*	Chi sq.	*P*
Survival					
	*L. maackii*	215.771	**<0.001**	93.800	**<0.001**
	Deer	17.958	**<0.001**	38.536	**<0.001**
	Deer × *L. maackii*	2.012	0.156	0.298	0.585
		***F***	***P***	***F***	***P***
Browse					
	*L. maackii*	0.590	0.564	4.425	0.306
	Deer	72.497	**<0.001**	106.814	**<0.001**
	Deer × *L. maackii*	0.906	0.338	0.0291	0.863
Relative height growth					
	*L. maackii*	0.102	0.750	0.191	0.662
	Deer	43.996	**<0.001**	36.506	**<0.001**
	Deer × *L. maackii*	5.005	**0.025**	0.605	0.437
Relative basal diameter growth					
	*L. maackii*	2.034	0.150	4.355	**0.038**
	Deer	0.113	0.737	0.398	0.528
	Deer × *L. maackii*	1.047	0.306	1.055	0.304
Plant moisture stress					
	*L. maackii*	16.712	**<0.001**	5.6251	**0.0121**
	Deer	9.612	**<0.001**	1.5247	0.1868
	Deer × *L. maackii*	0.337	0.523	0.9183	0.3064
Foliar nitrogen					
	*L. maackii*	1.792	0.171	0.466	0.490
	Deer	0.512	0.474	0.321	0.571
	Deer × *L. maackii*	0.336	0.562	2.292	0.130
**Environmental Variables**		***F***	***P***		
Soil nitrogen	*L. maackii*	0.047	0.832		
	Deer	1.885	0.195		
	Deer × *L. maackii*	0.522	0.484		
PAR	*L. maackii*	73.607	**<0.001**		
	Deer	0.089	0.766		
	Deer × *L. maackii*	0.000	0.987		

### Seedling characteristics and browse

**Figure 2.**
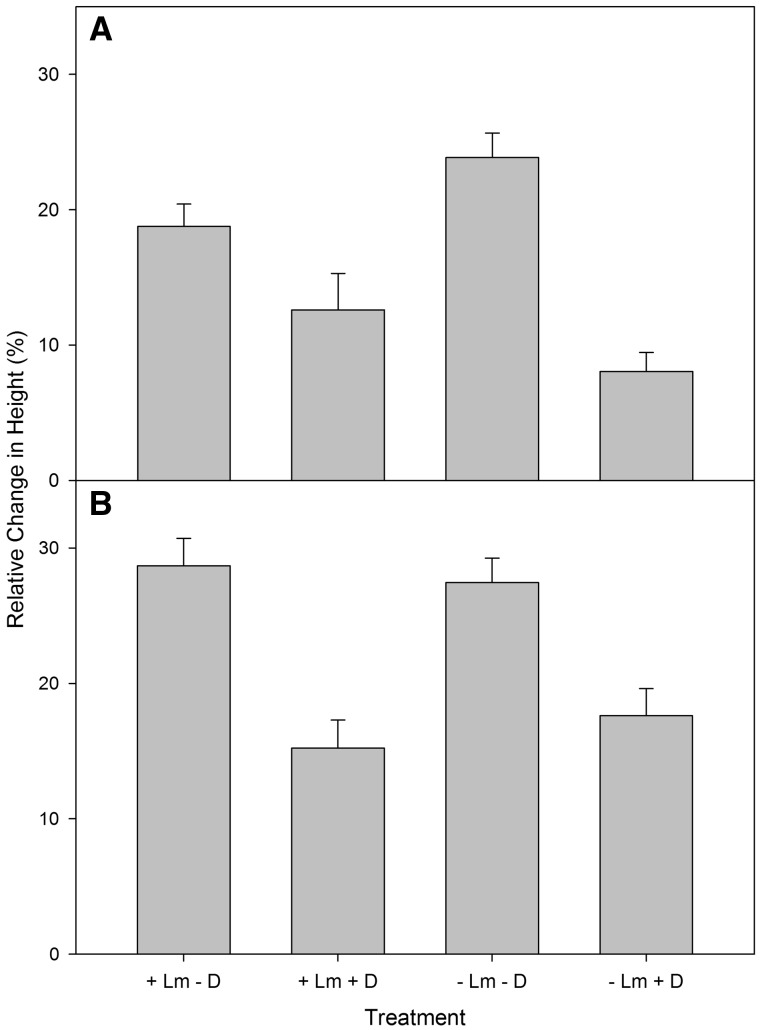
Relative change in height (%) of underplanted seedlings of (A) *C. dentata* and (B) *Q. rubra* with *L. maackii* (+ Lm) and where it was removed (- Lm) and where deer had access (+ D) and where they were excluded (- D). Data are mean ± 1 SE.

The presence or absence of *L. maackii* outside of the deer exclosures did not significantly impact the number of underplanted seedlings of either species browsed by the end of the study, a finding that did not support our prediction that *L. maackii* would protect seedlings from browsing (Table 2). We observed a significant difference in relative height during the first growing season, with white-tailed deer having a negative effect on growth in height for seedlings of both species (Fig. 2, Table 2). While *L. maackii* did not have a significant effect on height for *Q. rubra*, there was a significant interaction between *L. maackii* and deer on height for *C. dentata*, with greater height growth in the treatment without *L. maackii* and deer (Table 2), supporting our prediction that seedlings would grow best when deer and *L. maackii* were absent*.* However, the next greatest height growth occurred in the treatment with *L. maackii* and without deer (Fig. 2, Table 2) and the least growth in height for *C. dentata* occurred in the two treatments in which deer had access (Fig. 2, Table 2), countering our prediction that *L. maackii* would have greater effects on height growth than deer.

**Figure 3.**
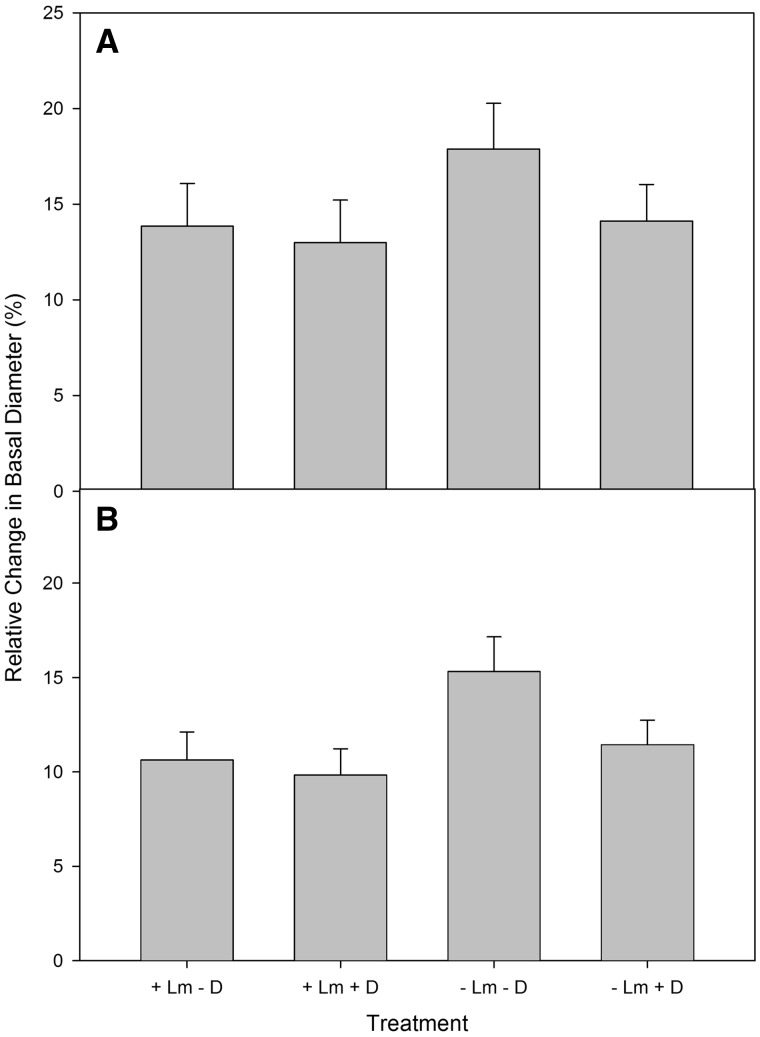
Relative change in basal diameter (%) of underplanted seedlings of (A) *C. dentata* and (B) *Q. rubra* with *L. maackii* (+ Lm) and where it was removed (- Lm) and where deer had access (+ D) and where they were excluded (- D). Data are mean ± 1 SE.

Deer did not have an effect on growth in basal diameter for either underplanted species. There was no difference amongst treatments in relative change in basal diameter for *C. dentata* (Fig. 3). For *Q. rubra*, relative change in basal diameter was greater in the areas where *L. maackii* was present (Fig. 3). The high mortality of seedlings in some of the treatments during the second growing season did not allow us to examine changes in basal diameter or height over the second growing season.

### Seedling moisture stress, PAR, and foliar and soil N

**Figure 4.**
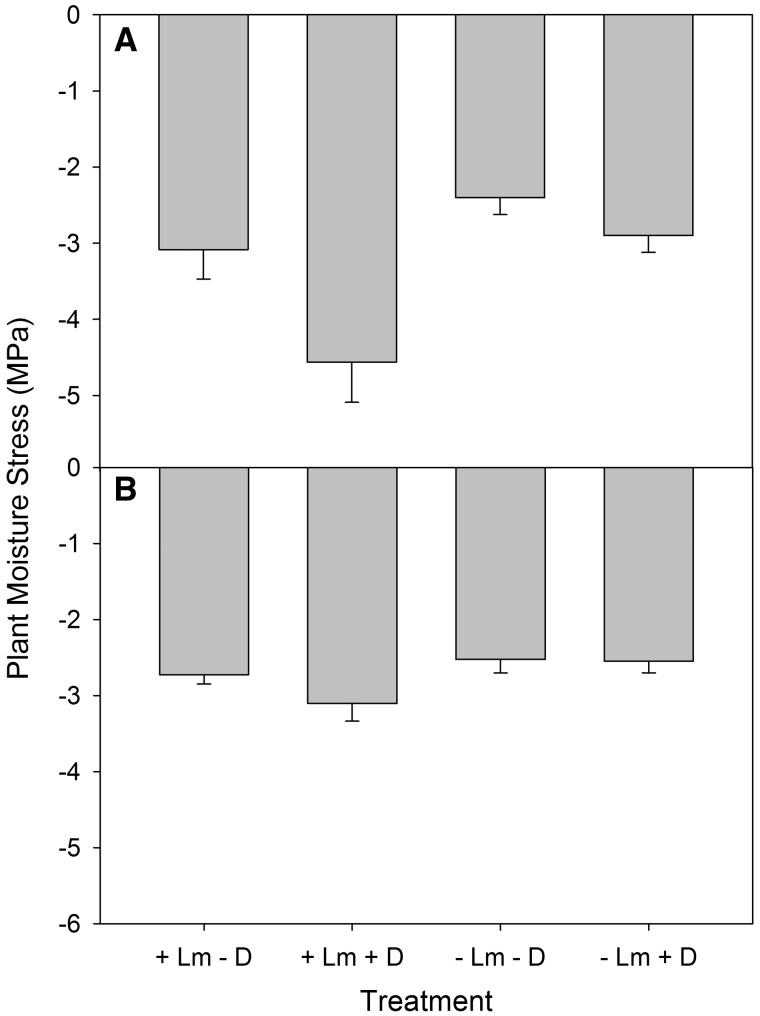
Plant moisture stress (MPa) for underplanted (A) *C. dentata* and (B) *Q. rubra* seedlings where *L. maackii* was present (+ Lm) and where it was removed (- Lm) and where deer had access (+ D) and where they were excluded (- D). Data are mean ± 1 SE.

Plant moisture stress was greater where *L. maackii* was present for both species of underplanted seedlings and outside the exclosures (deer present) for *C. dentata* (Fig. 4; Table 2). Percent ambient PAR was lower in areas in which *L. maackii* was present compared with where it was removed (Table 3). There was no difference in PAR between the exclosed and unexclosed areas within the *L. maackii* removal and reference areas (Tables 2 and 3). No significant difference was found in foliar nitrogen concentration amongst the treatments for either the *Q. rubra* or *C. dentata* underplanted seedlings (Table 2). There was also no significant differences in soil nitrogen amongst the treatments (Tables 2 and 3).

**Table 3:** Percent PAR (mean ± 1 SE) and percent soil nitrogen for study areas with *L. maackii* (reference), where *L. maackii* was removed (removal) and where deer had access (outside) and where they were excluded (inside). PAR measurements were recorded 1 m above the ground.

Treatment		% PAR (µmol/m^2^ s)	% N
Reference	Inside	1.450 ± 0.164	0.134 ± 0.010
Reference	Outside	1.716 ± 0.288	0.146 ± 0.014
Removal	Inside	2.802 ± 0.256	0.139 ± 0.011
Removal	Outside	3.419 ± 0.440	0.143 ± 0.013

### Natural regeneration

Contrary to our predictions, neither the presence of *L. maackii* nor white-tailed deer had an effect on the overall density of native tree seedlings across treatments. There was also no effect of *L. maackii* or white-tailed deer on the species richness, evenness, or diversity of naturally regenerated tree seedlings (Table 4). No invasive tree seedlings were encountered in our units.

**Table 4:** Generalized linear mixed models results for the effects model results for the effect of *L. maackii* (LM), white-tailed deer (D) and time (T; years) on the total density, species richness, species evenness and species diversity of naturally regenerating native tree seedlings and the density of the most common species across the five sites. Values represent *P*-values for the main effects and main effect interactions with values in bold representing effects that were significant (*P* < 0.05). *P*-values for density were adjusted for multiple comparisons with a graphically sharpened procedure to control the false discovery rate (Benjamini and Hochberg 1995).

	LM		D		T		LM*D		LM*T		D*T		LM*D*T	
	*F*	*P*	*F*	*P*	*F*	*P*	*F*	*P*	*F*	*P*	*F*	*P*	*F*	*P*
Seedling density	0.61	0.44	0.13	0.72	0.13	0.71	0.02	0.90	1.95	0.17	1.57	0.21	2.93	0.09
Species richness	1.25	0.28	0.06	0.82	0.23	0.69	1.94	0.16	0.05	0.87	0.79	0.37	0.21	0.65
Species evenness	2.34	0.13	2.21	0.14	1.44	0.23	2.44	0.12	0.69	0.41	0.27	0.60	1.65	0.20
Species diversity	2.66	0.10	0.35	0.55	0.95	0.33	3.44	0.06	0.85	0.36	0.07	0.78	0.68	0.41
*Acer saccharum*	59.71	**0.005**	0.09	0.81	11.36	0.26	2.22	0.40	0.01	0.83	1.25	0.30	1.47	0.52
*Carya* spp.	0.39	0.64	1.22	0.64	5.32	0.62	0.12	0.76	0.20	0.73	2.90	0.30	1.07	0.52
*Celtis occidentalis*	0.72	0.64	0.27	0.76	0.44	0.76	0.33	0.76	0.01	084	0.01	0.94	0.38	0.75
*Fraxinus americana*	0.21	0.83	8.15	0.04	0.27	0.64	010	0.76	22.95	**0.005**	10.26	**0.009**	0.01	0.84
*L. tulipifera*	0.63	0.64	0.49	0.73	0.58	0.71	0.75	0.76	4.35	0.13	3.74	0.26	0.06	0.82
*Prunus serotina*	10.55	**0.005**	0.70	0.64	11.91	**0.04**	0.01	0.83	2.01	0.40	0.13	0.77	0.24	0.76
*Quercus* spp.	0.40	0.76	0.27	0.76	0.02	0.81	0.03	0.83	0.02	0.83	1.84	0.44	0.18	0.76
*Sassafras albidum*	3.85	0.32	3.22	0.34	4.12	0.64	0.09	0.77	0.47	0.77	4.06	0.25	2.04	0.40
*Ulmus* spp.	5.66	0.34	2.08	0.49	0.47	0.34	12.80	**0.005**	6.62	0.36	7.10	**0.04**	0.53	0.70

Amongst individual species, the density of *Acer saccharum*, a highly shade-tolerant species, was greater where *L. maackii* was present than where it was removed (Tables 4 and 5, while the density of *P.serotina* was greater in the areas where *L. maackii* was removed (Table 5). Regardless of treatment, the density of *P. serotina* decreased over time. There was a significant interaction of *L. maackii* and time on the density of *F. americana*, with *F. americana* density decreasing over time in the *L. maackii* reference areas, but increasing in the removal areas. In contrast, the densities of *F. americana* and *Ulmus* spp. increased significantly outside of the exclosures over the course of the study and declined inside. The only significant interaction between white-tailed deer and *L. maackii* was for the density of *Ulmus* spp., which was greater in the treatment with *L. maackii* and white-tailed deer and did not differ amongst the other three treatments (Table 4).

**Table 5:** Density (stems/hectare ± 1 SE) of native tree seedlings across sites averaged across sampling periods from fall 2013 to 2015 in the treatments where *L. maackii* was present (*L. maackii*) and removed (no *L. maackii*). Superscript letters next to the species represent significant interactions between *L. maackii* and time (T) and deer (D). *P*-values for density were adjusted for multiple comparisons with a graphically sharpened procedure to control the false discovery rate (Benjamini and Hochberg 1995).

Density (stems/ha)			
Species	*L. maackii*	No *L. maackii*	*P*-value
*Acer saccharum*	8960 ± 1940	640 ± 200	0.005
*Carya* spp.	1120 ± 220	860 ± 198	0.64
*Celtis occidentalis*	1760 ± 260	2000 ± 280	0.64
*Fraxinus americana*^T^	26 360 ± 6780	29 280 ± 9480	0.83
*Liriodendron tulipifera*^T^	3460 ± 1080	3840 ± 920	0.64
*Prunus serotina*	4260 ± 920	7520 ± 1120	0.005
*Quercus* spp.	640 ± 200	580 ± 164	0.76
*Sassafras albidum*	620 ± 220	1520 ± 400	0.32
*Ulmus* spp.^D^	2680 ± 600	1440 ± 340	0.34

## Discussion

### Survival and growth of underplanted seedlings

Our finding that the presence of *L. maackii* and deer resulted in the lowest survival of underplanted seedlings of *C. dentata* and *Q. rubra* supported our hypothesis and the results of other studies that examined the individual effects of *L. maackii* and white-tailed deer and found each to have negative effects on the survival of naturally regenerating native tree seedlings (Loomis *et al.* 2015; Gorchov and Trisel 2003; Rossell *et al.* 2005). The increased survival of underplanted seedlings in areas in which *L. maackii* was removed and white-tailed deer excluded is likely due to a number of factors. In our study, percent ambient PAR was lower in the areas in which *L. maackii* was present. Both *Q. rubra* and *C. dentata* seedlings have intermediate tolerance of shade, but are intolerant of heavy shade (Crow 1988; Joesting *et al.* 2009). Thus, the reduced light levels in the *L. maackii* reference areas may have contributed to lower survival of the underplanted seedlings in the heavily shaded reference areas. Furthermore, reducing midstory basal area can promote growth and establishment of *Q. rubra* and *C. dentata* seedlings (Loftis 1990; Brown *et al.* 2014). The removal of *L. maackii* in our study may have had a similar effect resulting in increased light availability to the underplanted seedlings, especially at sites with high pre-removal densities of mature *L. maackii* (Table 1).

Overall, our results illustrated that, contrary to our prediction, the negative individual effects of deer on survival and height growth of underplanted seedlings were greater than those of *L. maackii*. One of the mechanisms by which white-tailed deer may have reduced survival of the underplanted seedlings is by reducing seedling height and photosynthetic tissue through browse. Other studies have found that height of naturally regenerating seedlings is greater in areas in which deer have been excluded or in areas with lower deer abundance (Horsley *et al.* 2003; Aronson and Handel 2011; Shelton *et al.* 2014). Gorchov and Trisel (2003) proposed that *L. maackii* may deter browsing of native seedlings by white-tailed deer. However, contrary to our prediction, we did not find a difference in the percent of underplanted seedlings browsed in non-exclosure areas by the end of the study. However, relative growth in height was greater with the exclusion of deer for both species of underplanted seedling, indicating that there was a negative effect of browsing on underplanted seedling growth. We observed no effect of *L. maackii* on relative growth in height for *Q. rubra*, but did find an interactive effect of *L. maackii* and deer on *C. dentata*, with underplanted seedling height being greatest where both were removed followed by where honeysuckle was present, but deer were excluded, indicating a stronger negative effect of deer. An effect of *L. maackii* on basal diameter was found for *Q. rubra* underplanted seedlings, with greater growth in areas in which *L. maackii* was removed. This finding may be a result of the higher PAR in the removal areas allowing for greater growth.

The lower survival of the underplanted seedlings in areas with deer and *L. maackii* may have been, in part, a result of increased seedling moisture stress in our study. Moisture stress of the underplanted seedlings was greater in the areas in which *L. maackii* was present for both species of underplanted seedlings, and for *C. dentata*, was greater in the areas accessible to deer. We predicted that moisture stress would be greater in areas with *L. maackii*, which reduces soil moisture and throughfall, potentially resulting in less water available to seedlings (Pfeiffer and Gorchov 2015). Precipitation during the two weeks prior to our measurements was almost twice the normal amount (U.S. Climate Data 2015), potentially having an impact on our results as underplanted seedlings may not have been under as high of stress as that which may typically occur under drier conditions. Despite this increased rainfall, we still detected differences in moisture stress, suggesting that deer may be increasing moisture stress of the underplanted seedlings through herbivory. The increased moisture stress created by the presence of *L. maackii* and deer likely has a stronger effect on mortality and growth during the periodic meteorological droughts that occur in the Midwestern United States (Mallya *et al.* 2013).

### Natural regeneration

Contrary to our predictions, neither *L. maackii* nor white-tailed deer had a significant effect on the overall density, richness or diversity of naturally regenerating native seedlings. In contrast, other studies have found reduced native tree seedling density, richness, diversity and survival under *L. maackii* (Collier *et al.* 2002) and in areas in which white-tailed deer are present (Tilghman 1989; Rossell *et al.* 2005; Aronson and Handel 2011; Frerker *et al.* 2014). While both *L. maackii* and white-tailed deer can individually have negative effects on native tree seedling survival and density, Gorchov and Trisel (2003) suggested that there may be an interaction between *L. maackii* and white-tailed deer in which *L. maackii* protects seedlings from browse and increases their survival. However, we found no interactive effect of white-tailed deer and *L. maackii* on overall seedling density, richness or diversity in our study.

The short time period in which our exclosures were in place may not have allowed us to detect changes in overall density and diversity resulting from deer herbivory. While *L. maackii* was removed from the sites up to three years prior to the beginning of the study, deer had only been excluded for ∼2.5 years by the end of the study. While changes in herbaceous-layer diversity richness and diversity have been found to occur quickly (1 year) after *L. maackii* removal (Shields *et al.* 2015b), changes in the herbaceous layer following deer exclusion may take longer to manifest (10+ years) and may not be detectable over shorter periods (Griggs *et al.* 2006; Collard *et al.* 2010; Frerker *et al.* 2014; Waller 2014). In addition, while deer population densities in our study areas were likely representative of densities across much of the Midwest, our sites do not have the long history of heavy overabundance documented in studies from other regions (Horsley *et al.* 2003; Griggs *et al.* 2006). Therefore, changes across our sites following deer exclusion may be subtler and slower to develop. However, the high rate of browse on the underplanted seedlings in our study shows that deer are affecting regeneration. Furthermore, the contemporary regeneration layer of forests across the Midwest reflects the cumulative effects of a regional deer population that is likely in excess of historic levels (Côté *et al.* 2004).

Other studies have observed suppressed regeneration as a result of lower light (Luken and Thieret 1994), allelopathy (Dorning and Cippollini 2006), and reduced soil moisture (Pfeiffer and Gorchov 2015) beneath *L. maackii.* In our study, the areas in which *L. maackii* was removed experienced large increases in the cover and height of herbaceous species (Freeman 2015), which may have reduced the survival of naturally regenerated seedlings through intensified competition as has been observed with natural regeneration of pine species (Cain 1991; Pitt *et al.* 2009, 2010).

While there was no effect of *L. maackii* or white-tailed deer on total native seedling density, richness or diversity, density and relative density differed for individual species and groups. *Prunus serotina* density was greater where *L. maackii* was removed, while *A. saccharum* density was lower in the absence of *L. maackii.*Shields *et al.* (2015b) found increases in *P. serotina* immediately following the removal of *L. maackii* across four of the sites in this study (our Martell site was not included in the study). While the high fecundity of this species allows it to produce high densities of seedlings, they are unlikely to grow out of the regeneration layer without canopy disturbance. While *A. saccharum* in our study may be able to reproduce under *L. maackii* cover, we did not assess the survival of individual seedlings. A recent study by Loomis *et al.* (2015) found no difference in *A. saccharum* survival after one year between plots where *L. maackii* was present and where it was removed, suggesting that the high shade tolerance of the species may allow it to persist under *L. maackii* cover. While we did not observe significant effects of deer or *L. maackii* on seedlings of *Quercus* species, densities of this genus were low across our study sites and likely precluded our ability to detect differences.

Through time, the density of two species groups, *Ulmus* spp. and *F. americana*, decreased inside the exclosures while increasing outside. While *F. americana* is browsed by deer (Tilghman 1989), the species can remain abundant in the presence of white-tailed deer and may be more tolerant of browse than many other tree species, allowing its density to remain high outside of the exclosures (Rossell *et al.* 2005; Jenkins *et al.* 2014). Similar to *F. americana*, the density of *Ulmus* spp. was greater outside the exclosures than inside (Table 4). Density of this species displayed a significant interaction between *L. maackii* and deer, with greater relative density in the presence of both *L. maackii* and deer. In areas of high deer populations, *Ulmus* spp. can be a frequently browsed species (Pogge 1967); however, in areas of lower deer abundance, it may be avoided when other more palatable species are available (LaGory *et al.* 1985; Sotala and Kirkpatrick 1972).

## Conclusions

Tree recruitment is a critical mechanism for the maintenance of ecological resilience in forests (Reyer *et al.* 2015). However, maintaining successful regeneration of native forests has become increasingly difficult in this era of global change. Shifts in the historical abundance of browsing ungulate populations and spread of invasive plant species are frequently identified as paramount challenges to forest regeneration worldwide, particularly in fragmented landscapes.

As in many other forests, the effects of ungulate herbivory, invasive plants and altered disturbance regimes were highly evident across our study sites. We observed natural regeneration layers that were dominated by late seral and browse tolerant species. The presence of *L. maackii* favoured only the most shade tolerant species (*A.saccharum*) and the added effects of deer pushed seedling-layer composition towards *F. americana* and *Ulmus* spp. However, *F. americana* and *Ulmus* spp. are unlikely to persist in the future canopy of these forests due to the effects of introduced insects and disease (Lovett *et al.* 2016). Under these conditions of depauperate natural regeneration, underplanting offers a potential, albeit expensive, technique to begin the restoration of desired species to degraded forests. Our study found that the removal of *L. maackii* increased understory light levels in a way similar to a technique offered by Loftis (1990) to regenerate oak species on mesic sites. This technique mechanically reduces the density of the midstory to increase light levels and foster the survival and growth of advance regeneration, which is ultimately released when canopy openings are created. Using a similar sequence, *L. maackii* removal, in conjunction with underplanting, could be a first step in restoring degraded hardwood forests. However, our results showed that the presence of deer had a greater negative effect on the survivorship of *C. dentata* seedlings than shading by *L. maackii.* The low survival of both underplanted species in the presence of deer and *L. maackii* demonstrates that both deer and *L. maackii* must be managed if restoration efforts are to be successful.

## Sources of Funding

This research was supported with funds from the Department of Forestry and Natural Resources and the Hardwood Tree Improvement and Regeneration Center at Purdue University and the USDA McIntire-Stennis Cooperative Forestry Program (project IND011533MS).

## Contributions by the Authors

This article was derived from the MS thesis of C.F.O., who was co-advised by D.F.J. and M.A.J. Study design was a collaborative effort of D.F.J., J.M.S., M.R.S. and M.A.J. C.F.O. performed the analyses and led the writing effort. D.F.J. and M.A.J. contributed text and all authors edited the manuscript.

## Conflicts of Interest Statement

None declared.
